# Multimodal Early Alzheimer’s Detection, a Genetic Algorithm Approach with Support Vector Machines

**DOI:** 10.3390/healthcare9080971

**Published:** 2021-07-31

**Authors:** Ana G. Sánchez-Reyna, José M. Celaya-Padilla, Carlos E. Galván-Tejada, Huizilopoztli Luna-García, Hamurabi Gamboa-Rosales, Andres Ramirez-Morales, Jorge I. Galván-Tejada, on behalf of the Alzheimer’s Disease Neuroimaging Initiative

**Affiliations:** 1 Unidad Académica de Ingeniería Eléctrica, Universidad Autónoma de Zacatecas, Jardín Juárez 147, Centro Historico, Zacatecas 98000, Mexico; ing.agsreyna19@gmail.com (A.G.S.-R.); jose.celaya@uaz.edu.mx (J.M.C.-P.); ericgalvan@uaz.edu.mx (C.E.G.-T.); hlugar@uaz.edu.mx (H.L.-G.); hamurabigr@uaz.edu.mx (H.G.-R.); 2 Department of Physics, Kyungpook National University, 80 Daehak-ro, Daegu 41566, Korea; andres@knu.ac.kr

**Keywords:** Alzheimer’s disease, support vector machine, genetic algorithm

## Abstract

Alzheimer’s disease (AD) is a neurodegenerative disease that mainly affects older adults. Currently, AD is associated with certain hypometabolic biomarkers, beta-amyloid peptides, hyperphosphorylated tau protein, and changes in brain morphology. Accurate diagnosis of AD, as well as mild cognitive impairment (MCI) (prodromal stage of AD), is essential for early care of the disease. As a result, machine learning techniques have been used in recent years for the diagnosis of AD. In this research, we propose a novel methodology to generate a multivariate model that combines different types of features for the detection of AD. In order to obtain a robust biomarker, ADNI baseline data, clinical and neuropsychological assessments (1024 features) of 106 patients were used. The data were normalized, and a genetic algorithm was implemented for the selection of the most significant features. Subsequently, for the development and validation of the multivariate classification model, a support vector machine model was created, and a five-fold cross-validation with an AUC of 87.63% was used to measure model performance. Lastly, an independent blind test of our final model, using 20 patients not considered during the model construction, yielded an AUC of 100%.

## 1. Introduction

Alzheimer’s disease is one of the most common neurodegenerative diseases, mainly affecting older adults. According to the World Health Organization [1] and Alzheimer’s Disease International [2], in 2018, dementia affected approximately 50 million people worldwide, with an estimate of 75 million by 2030 and approximately 150 million by 2050. Moreover, there is a co-occurrence of AD with several chronic diseases, such as diabetes mellitus, which aggravate the treatment and outcome [3].

Although Alzheimer’s disease has no cure, there are pharmacological treatments to control the symptoms. Diagnosing AD in the mild stages allows the use of treatments that might delay the progression of the disease. Late AD detection, however, may lower the effectiveness of a given treatment. Hence, early detection is imperative for maximum efficiency [4]. There are efforts devoted to study Alzheimer’s disease, such as the Alzheimer’s Disease Neuroimaging Initiative (ADNI) [5], which has documented a database of medical images, data from biological markers (biomarkers), and clinical and neuropsychological assessments of patients since 2004; these data are publicly available for scientific research.

Typically, biomarkers to diagnose AD are extracted from the analyses of medical images such as MRI and PET [6,7]. Moreover, blood metabolites have been studied as possible biomarkers in the AD diagnosis [8]. Others techniques utilize speech data, which contain features extracted from the spectrogram of the patient’s voice [9]. Recently, the combination of clinical and neuropsychological assessments to extract biomarkers has attracted attention, since these assessments are economic, easy to apply and compute their effectiveness and are accessible in places where blood and medical images tests are difficult to find [10,11,12]. These characteristics make the clinical and neuropsychological assessments useful for early AD detection. This paper focuses on the latter assessments.

Several medical investigations are based on the use of multivariate models, that is, the use of multiple features (biomarkers in this work) and their correlation. Through machine learning (ML), artificial intelligence (AI) assists in creating a multivariate model that aims to describe a given disease; the model is trained/fitted using well-characterized data, empowering the model to infer properties of data not considered during the training phase. In recent years, the use of ML in the area of medicine has played an important role in the diagnosis, prediction and classification of diseases. ML has improved the processing of medical images from different specialties and diagnosing with good precision various diseases such as breast cancer, skin cancer, colon cancer, cerebral microbleeds, diabetes disease and cardiovascular disease, in conjunction with others [13,14,15]. In the present work, the use of ML for early AD detection, is explored.

One of the main challenges in the development of new biomarkers, for multifactorial diseases, is the reduction of the data dimensionality. Copious sources of information, such as clinical, imaging, metabolomic, etc., are readily available; the latter requires great efforts to generate methodologies that reduce the number of features/dimensionality for the efficient development of biomarkers. A promising approach to tackle this dimensionality reduction challenge is the use of genetic algorithms. The genetic algorithms are techniques of evolutionary computation, with low computational requirements, for finding solutions to complex search and optimization problems [16]. Inspired by the Darwinian theory of evolution, a genetic algorithm evolves iteratively a population of chromosomes (solutions) and their genes, through a process of selection, crossover, and mutation, where the fittest solutions (best biomarkers in the present case) prevail.

**Figure 1:**
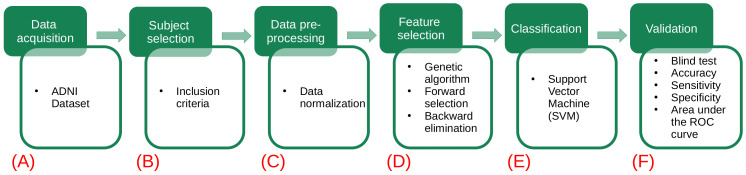
Flowchart of the proposed methodology. The green squares refer to the data processing methodology, while the white squares detail the task involved in each step. (**A**) The different datasets (gene indexes and clinical and neuropsychological assessments) are obtained from the ADNI database. (**B**) Each dataset is analyzed and new data sets are created by selecting subjects according to the criteria described in Table 2. (**C**) A preprocessing of the data is applied: handle the empty fields and perform data transformations. (**D**) The use of genetic algorithms is implemented to extract the main data features. (**E**) Using the main features for Alzheimer’s detection in patients, several models are generated using the support vector machines. (**F**) The validation of our results is carried out using different metrics (accuracy, sensitivity, specificity and AUC) to determine which of the models has the best performance.

Specifically, this work proposes the use of ADNI-related features, gene indexes, and clinical and neuropsychological assessments as features to build ML models based on support vector machines to describe AD. Support vector machines are chosen as they offer high accuracy and work well in high dimensional spaces [17]. In addition, we propose the use of a genetic algorithm to select the most robust models to obtain biomarkers for the detection of AD at an early stage of the disease between MCI and cognitive normal patients. We expect that the combination of genetic algorithms and support vector machines will benefit the early detection of AD. This paper is organized as follows: Section 2 discusses the criteria that could be used to diagnose AD and addresses related work. Section 3 describes the methodology used to build a model to classify subjects with AD vs. MCI and cognitive normal (CN) (see Figure 1). Section 4 presents the results obtained from the models, while Section 5 addresses the discussion, where the results of the final model are presented and compared with other studies, a zoomed view of the figures for the results of this section can be found in Appendix A. Finally, Section 6 and Section 7 present the conclusions and future work, respectively.

## 2. Alzheimer’s Disease Diagnosis and Related Work

### 2.1. Alzheimer’s Disease Diagnosis

It is possible to diagnose AD by combining clinical and neuropsychological assessments, in conjunction with medical imaging. For example, the analysis of cerebrospinal fluid (CSF) biomarkers with respect to neuropsychological assessments is used to determine the degree of degeneration of cognitive and behavioral functioning. This class of studies encloses the Mini-Mental State Examination (MMSE) [18], which is a set of standardized questions used internationally to measure cognitive impairment. The MMSE score is calculated by tallying the number of questions answered correctly; a lower score indicates greater cognitive impairment. The Alzheimer’s Disease Assessment Scale (ADAS) Cognitive Subscale with 11 items (ADAS-Cog 11) [19] and its variant with 2 additional items (ADAS-Cog 13) [20] are sub-scales of the ADAS for differentiating between normal and impaired cognitive functioning and assessing the severity of cognitive symptoms of dementia. The Geriatric Depression Scale (GDS) [21], which is a test that indicates the presence of depression in older adults, and the Functional Activities Questionnaire (FAQ) [22], which measures functional changes in adults using a scale of instrumental activities, are also key assessments in diagnosing AD. Additionally, there are estimations for the global scaling of dementia that clinically evaluate and classify its progression and severity, such as the Global Deterioration Scale [23], which assesses the degree of deterioration of cognitive function, and the Clinical Dementia Rating (CDR), which is a staging instrument for classifying the severity of dementia. The CDR produces a global score (CDGLOBAL) that determines the stage of dementia and a sum-of-boxes score (CDRSB) that measures the severity of dementia. An algorithm is used to calculate the CDGLOBAL score, while the CDRSB score is calculated by summing each of the domain box scores [24] (see Table 1). In terms of medical imaging, magnetic resonance imaging (MRI) and positron emission tomography (PET) are the most commonly used. This work studies clinical and neuropsychological assessments and laboratory analysis for the diagnosis of AD. Some considered examples are listed in Table 1.

### 2.2. Related Work

Machine learning methodologies have been applied successfully in the context of the study of AD. For example, Daoqiang Zhang et al. [6] built an ML model by combining: ADNI baseline features from three modalities, using data from MRI (to measure brain atrophy), hypometabolism, and certain CSF proteins to classify patients with AD (or MCI) vs. CN. They used a multiple-kernel support vector machine [25] model to classify patients, resulting in an accuracy of 93.2%, a sensitivity of 93% and a specificity of 93.3% to classify AD vs. CN. To classify MCI vs. CN, a classification accuracy of 76.4%, a sensitivity of 81.8% and a specificity of 66% were reported. Hassan et al. [26] combined non-imaging biomarkers, a CSF biomarker and clinical data to generate and compare three ML models. The goal was to classify CN vs. MCI patients. The best ML model, was the J48 decision trees [27], classifying the patients with an accuracy of 96.92%, area under the receiver operating characteristic curve (AUC) of 0.985, sensitivity of 100% and specificity of 95.74%. More recently, in 2019, Stamate et al. [28], conducted a study with clinical and cognitive data in combination with blood metabolite data for the classification of CN vs. AD patients. Three different ML models were compared, obtaining XGBoost [29] as the best model with an AUC of 0.88.

## 3. Methodology

The proposed methodology for this study consists of six stages, as shown in Figure 1. In the first stage, the used datasets are described (Figure 1A). In the second stage, the dataset of interest is created by selecting the subjects according to a given inclusion criteria (Figure 1B). In the third stage, data preprocessing is applied, and verification and treatment of the empty fields and data transformation are performed (Figure 1C). In the fourth stage, feature selection is implemented by means of a genetic algorithm (Figure 1D). Then, a representative set of biomarkers are studied using support vector machine classifiers (Figure 1E). Finally, a validation test is done considering different metrics (accuracy, sensitivity, specificity and AUC) to determine the performance of our model (Figure 1F).

### 3.1. ADNI Database

The data used in this study were obtained from the ADNI database (adni.loni.usc.edu; accessed on 7 September 2020). The ADNI was launched in 2003 as a public–private partnership, led by the Principal Investigator Michael W.Weiner, MD. The primary goal of ADNI has been to test whether serial MRI, PET, other biological markers, and clinical and neuropsychological assessments can be combined to measure the progression of MCI and early AD. For up-to-date information, see www.adni-info.org; accessed on 7 September 2020.

### 3.2. Data Selection

An exhaustive analysis of the ADNI database was carried out, and more than 200 datasets were analyzed, containing over 314 observations and 2279 features. A filter was implemented to determine which of these features may be used in the proposed study. A reduced dataset was generated with only those patients and features that met the conditions indicated in Table 2, the dataset called “upennbiomk3” was taken as the initial dataset [5,30], which has information of 106 patients from the ADNI1 study with two or more visits each. The objective of this study is to analyze and observe the relationship of the data for the classification and/or diagnosis of the disease.

The resulting filtered dataset (FDS), after applying the above inclusion criteria of Table 2, contains information corresponding to 106 patients (42 Female/64 Male), age (75.95 ± 6.02), clinical and neuropsychological evaluations and diagnoses (CN = 36, MCI = 52, AD = 18).

#### Data Preprocessing

The FDS dataset, fulfilling the visit code “bl” requirement, comprises 103 observations (42 Female/61 Male) and excludes 3 patients lacking this visit code. For the qualitative features, a nominal scale was made; thus, the feature used for the diagnosis of the patient (DX) remained as a binary variable. When the patient’s diagnosis is CN or MCI, a “0” label was assigned, otherwise, if the patient’s diagnosis is AD, a “1” was assigned. Once the dataset was composed of only numerical variables, a filter was performed to drop features missing more than 6.8% of the values. Thus, the final dataset size consists of 103 observations and 927 features. From this point, two versions of FDS were ccreated under the following criteria:

- Dataset 1 (D1): The missing values in a given feature were substituted with the mean value of this feature to complete the records (103 observations and 927 features).
- Dataset 2 (D2): From the above dataset D1, the neuropsychological features are eliminated, leaving a grand total of (103 observations and 904 features).

In D2, neuropsychological features were removed in an attempt to find new features that would aid in the diagnosis of AD, given these neuropsychological features were found to have a high correlation with AD during the experimentation described in Section 3.3.1.

Subsequently, the datasets were scaled to transform their features. In this case, a z-score transformation was applied, that is, the mean and the standard deviation of each feature are transformed to zero and one, respectively. The transformed values, $z_{i}$, are expressed as [31],

(1) $$z_{i}=\frac{x_{i}-\bar{x}}{\sigma },$$

where $x_{i}$ are the raw values, $\bar{x}$ is their mean and σ is their standard deviation of each feature in the dataset.

Finally, for both D1 and D2, 80% of the data was used for training and testing, and the remaining (20%) was saved for an independent blind test.

### 3.3. Model Generation

ML is a part of AI, which allows statistical models to learn from the interaction between the input data and their processes [32], achieving the identification of complex patterns within the data to classify, identify, optimize or predict future behaviors. ML models learn through previous experience and extraction of generic knowledge from data, being able to improve themselves autonomously, achieving excellent performance in making predictions. With this motivation, this paper describes a methodology that aims to generate ML models that allow the detection of AD at an early stage. Therefore, it is necessary to select features in D1 and D2 that help build a robust model that combines information from different sources such as gene indexes and clinical and neuropsychological assessments.

**Figure 2:**
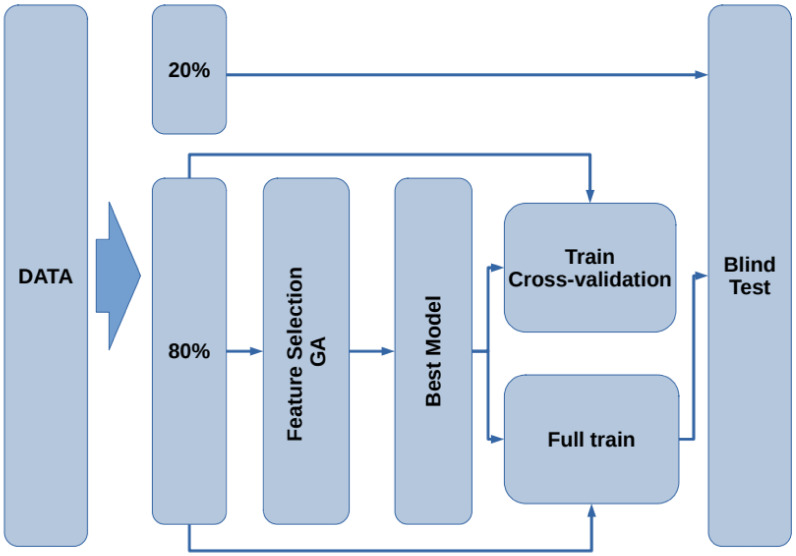
Flowchart of the proposed methodology for the model generation and validation.

The flow chart for the model generation is presented in Figure 2. First, the data are split into two subsets containing 80% of the data for training and testing, leaving the remaining 20% for blind testing. Using the 80% subset, the feature selection procedure is performed by means of a genetic algorithm. Next, with the best selected features, a model that describes the data is generated. To assess the train/test performance of this model, a k-fold cross-validation [33] is carried out. Later, using the whole 80% subset, the model is trained to generate the final model. Finally, the remaining 20% of the data (blind dataset) is used as unseen samples to assess the performance of the model on new subjects. In the following subsections, each stage is presented.

#### 3.3.1. Feature Selection

Due to the large number of features (more than 900) in the datasets D1 and D2, constructing models, which are capable of solving classification problems, becomes a very complex computational task. Therefore, the use of genetic algorithms is proposed. Namely, the GALGO [34] genetic algorithm (GA) is employed here, since it is efficient for selecting the best subset of features in high dimensional datasets. For this study, GA creates an initial population of chromosomes constituted of random sets of features. The fitness of the chromosomes is evaluated by comparing their ability to correctly detect AD subjects. Depending on the obtained fitness score, the process stops if the chromosome score is higher than the predefined goal and this chromosome is selected. On the other hand, if the process continues, the chromosome population is replicated and the chromosomes crossover and mutate; in this manner, the fittest chromosomes will produce next generation offspring. This step is repeated until a chromosome is found that meets the previously established criteria. The considered GA parameters are described in Table 3.

The GA was used to select the best subset of features from the FDS. The GA fitness of the chromosomes is calculated, employing support vector machines as binary classifiers. The support vector machine (SVM) model, introduced by Vladimir Vapnik [25], was chosen since it is robust and could be used to solve binary classification ML problems. The SVM model uses the theory of Structural Risk Minimization to maximize its prediction accuracy and procures avoiding data overfitting [35]. The SVM classification is carried out by mapping the original feature space, with kernel functions, to a hyperspace where a hyperplane is constructed, which separates the data of one class from the other [25,34].

**Figure 3:**
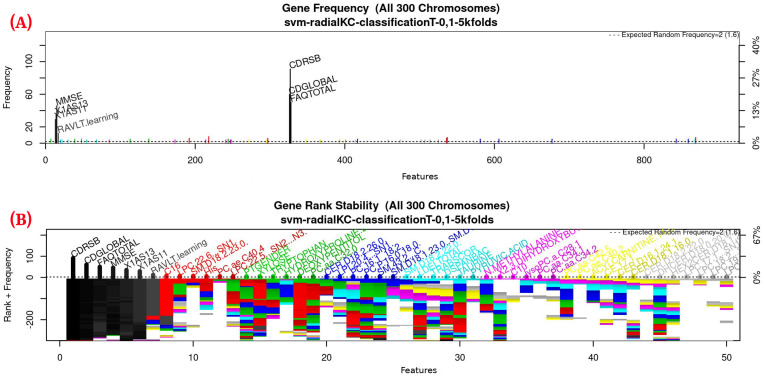
Gene frequency and rank in the models determined by implementing GA using the parameters in Table 3 for the selection of the top features in the dataset. (**A**) Gene frequency shows the number of times that a feature has been present in the models. (**B**) Gene rank shows the stability and frequency of each feature within the models, ordered by rank. For a zoomed view see Figure A1.

**Figure A1:**
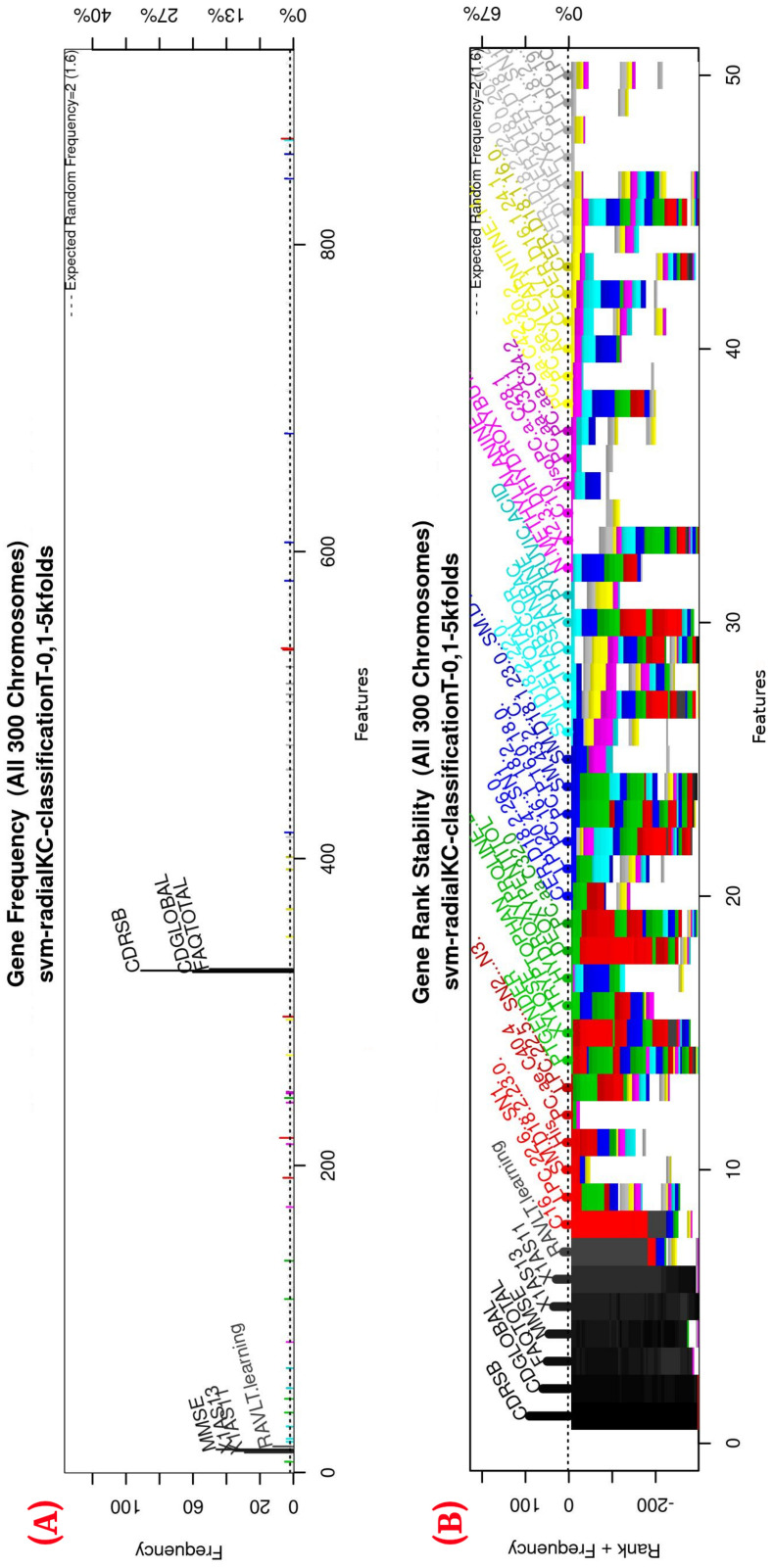
Gene frequency and rank in the models determined by implementing GA using the parameters in Table 3 for the selection of the top features in the dataset. (**A**) Gene frequency shows the number of times that a feature has been present in the models. (**B**) Gene rank shows the stability and frequency of each feature within the models, ordered by rank.

For this study, an SVM [36] with a radial kernel was used as a classification method in the feature genetic search; the specific parameters of the model are shown in Table 3. The top-50 most frequent features are obtained. This ranking is then used in the next step to build the final model. The features appearing more frequently in this selection suggest that they are of importance to the classification of AD patients, see Figure 3 (see Figure A1 for a zoomed view). Subsequently, a model refinement was carried out by means of forward selection and backwards elimination to select the most compact and accurate model (see Table 4) [37].

**Table 3:** GA input parameters. The genetic selection parameters are as follows: The chromosome size is set according to the recommendation found in Reference [34]. The number of solutions is defined to avoid bias. The number of generations is set to allow most of the models to converge (see Figure 4 or Figure A2 for a zoomed view). The goal fitness is defined to obtain a minimum performance required. The SVM hyper-parameters are as follows: The cost *C* is set to control the trade-off between decision and classification error and to avoid overfitting. A small γ value restricts the curvature of the decision boundary. A radial basis function is selected as the SVM kernel since it yields good out-of-box performance [38].

	Parameter	Value
Genetic selection	Classifier	SVM
	Chromosome size	5
	Max solutions	300
	Max generations	200
	Goal fitness	0.9
SVM	Cost *C*	1
	Gamma γ	0.2
	Kernel	Radial

The forward selection algorithm creates models by adding one feature at a time and keeps this feature in the model only if it contributes to the overall model accuracy. The forward selection process generates models that allow obtaining a high level of classification accuracy; however, this process can add a large number of features, which could overfit the data. To avoid the latter, a backwards elimination process was carried out; in this process, one feature is removed one at a time if the performance does not drop considerably. On the other hand, if the performance of the model decreases, the feature is kept. Figure 5 (see Figure A3 for a zoomed view) shows the performance of the models obtained during the forward selection process; for more details, please refer to the results in Section 4.

### 3.4. Model Training and Validation

Once the feature selection process was completed, a SVM classification model was created to study D1 and D2 containing only the best features; a linear kernel was chosen given its simplicity and expected good performance [39]. The SVM model was validated following a five-fold cross-validation strategy on the 80% dataset (see Table 5), and the best penalty cost for each model was found to be C=1 for the ADvsMCI/CN-m1 model and C=10 for the ADvsMCI/CN-m2 model. Next, using the whole 80% training dataset, the model was fitted and used as the final model. Lastly, with the final model, a blind validation test was performed on the 20% blind dataset in order to measure its correctness in diagnosing AD in new unseen subjects (see Table 6), allowing to simulate a real-life scenario.

### 3.5. Performance Analysis

The performance of these models was measured through the classification metrics: accuracy, sensitivity and specificity (see Table 6). These metrics establish which of the models is the best for identifying Alzheimer’s patients and which features are the most significant to obtain the best results in each phase. Sensitivity, defined in Equation (2), refers to the correct identification of patients with dementia (true positive). Specificity, defined in Equation (3), refers to the correct identification of patients without dementia (true negative). Accuracy is the percentage of cases that the model has classified correctly and is defined in Equation (4).

(2) $$Sensitivity=\frac{T_{p}}{T_{p}+F_{n}}$$

(3) $$Specificity=\frac{T_{n}}{T_{n}+F_{p}}$$

(4) $$Accuracy\left(1-Error\right)=\frac{T_{p}+T_{n}}{T_{p}+T_{n}+F_{p}+F_{n}}$$

where

*T_p_* = True positive, number of subjects with dementia correctly classified
*F_p_* = False positive, number of healthy subjects incorrectly classified.
*T_n_* = True negative, number of healthy subjects correctly classified.
*F_n_* = False negative, number of subjects with dementia classified as healthy.

The AUC [40] has been used to measure the performance of a classifier as well. The AUC describes how good a model is at making a prediction, and the AUC value ranges from 0 to 1; 0 for an incorrect prediction of 0% and 1 for a 100% correct prediction. This metric is computed with the sensitivity and specificity. The simplest way to calculate the AUC is to use trapezoidal integration [40].

## 4. Results

The obtained models and the classification metrics are presented in Table 4, Table 5 and Table 6. It is observed that the ADvsMCI/CN-m1 model and the ADvsMCI/CN-m2 model performed equally in the blind test. This test reproduces the conditions in a real-life scenario to diagnose AD in new unseen patients. Consequently, to choose the best model, an additional comparison of the length of the models, their features and the method of calculating their scores (MMSE, CDRSB, CDGLOBAL), was performed: The ADvsMCI/CN-m1 model contains only two features, MMSE and CDRSB, and has scores that are easier to calculate than the CDGLOBAL assessment. Furthermore, in clinical and research areas, the MMSE and CDRSB are more widely used to stage the severity of dementia. Therefore, the ADvsMCI/CN-m1 model was established as the best performing model for classifying AD patients.

**Figure 4:**
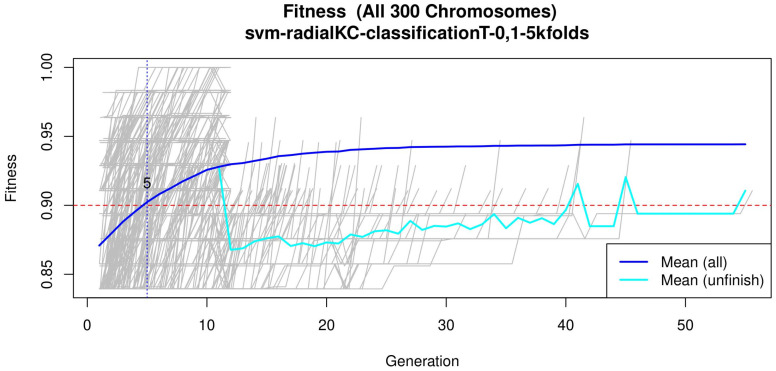
Evolution of the maximum fitness score across generations. The horizontal axis represents a given generation, whilst the vertical axis represents the fitness score. The average fitness, plotted with a blue solid line, considers all models. The average unfinished fitness, plotted with a cyan solid line, considers all searches that failed for a given generation and represents the average worst case expectation. The established GA goal fitness is plotted with the red dotted line. For a zoomed view see Figure A2.

**Figure 5:**
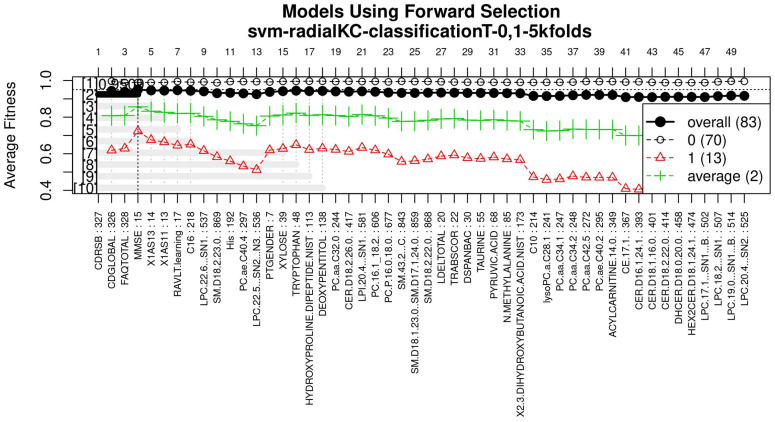
Performance of the most compact and accurate models after using the forward selection methodology. The horizontal axis represents the features ordered by rank. The vertical axis shows the classification accuracy. For a zoomed view see Figure A3.

Figure 3, Figure 4 and Figure 5 (see Appendix A for a zoomed view), show the results obtained from the application of the GA considering the GA parameters in Table 3. The selected top features, for the development of the most representative model, are found by this GA configuration.

Figure 3 (see Figure A1 for a zoomed view) shows the results of the feature occurrences in the models. The horizontal axis in Figure 3A shows the features. The left-vertical axis shows the gene frequency, that is, the number of times a feature has been present in the models. The right-vertical axis shows the corresponding percentage in relation to the total number of models. Figure 3B shows the GA outcome rank stabilization, and this graph shows the frequency (vertical axis) of the best features found by the GA algorithm in a rank-descent fashion (horizontal axis), where the solid colors represent stable features that always aid in the classification. For a zoomed view of the figures, please refer to Appendix A and feature selection in Section 3.3.1 for more details. The inclusion procedure, applied to the FDS dataset, included 103 patients and 927 features. The feature selection was implemented using a GA, which evolved a total of 200 generations, and it was repeated 300 times. Figure 3 shows the stability ranking of the first 50 features found through this GA. The features are ordered from the most to the least frequent appearance.

**Figure A2:**
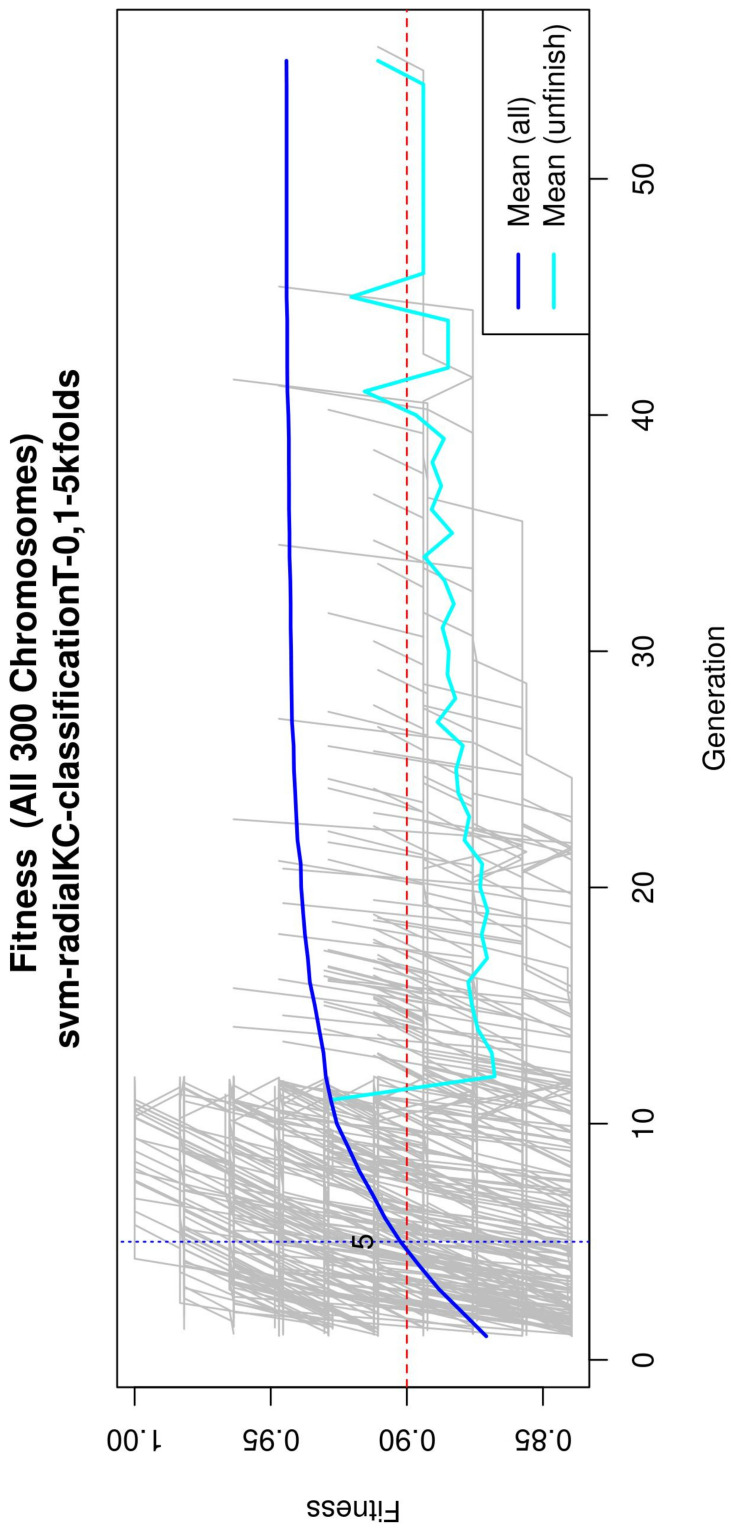
Evolution of the maximum fitness score across generations. The horizontal axis represents a given generation, whilst the vertical axis represents the fitness score. The average fitness, plotted with a blue solid line, considers all models. The average unfinished fitness, plotted with a cyan solid line, considers all searches that failed for a given generation and represents the average worst-case expectation. The established GA goal fitness is plotted with the red dotted line.

Figure 4 (see Figure A2 for a zoomed view) shows the fitness of the evolved models, where the blue line represents the mean fitness considering all models and the red line represents the generation in which the average fitness reaches the goal fitness. Analyzing this figure, it was determined that the GA parameters in (Table 3) are appropriate, since the number of generations needed to find an optimal model is less than 50 generations on average.

**Figure A3:**
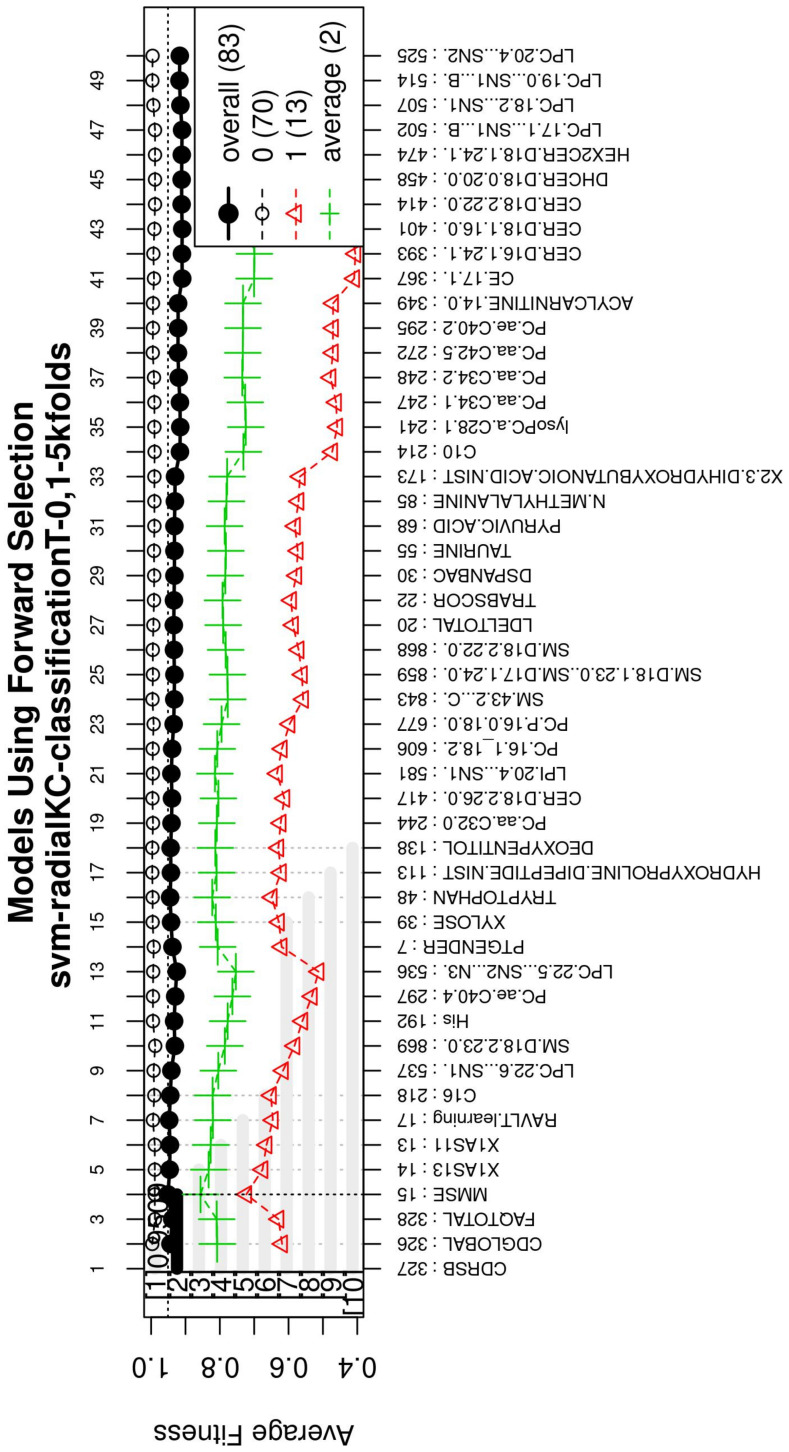
Performance of the most compact and accurate models after using the forward selection methodology. The horizontal axis represents the features ordered by rank. The vertical axis shows the classification accuracy.

With the ranked features (Figure 3), a forward selection procedure was used to create a representative model to classify AD vs. MCI/CN. Figure 5 (see Figure A3 for a zoomed view) demonstrates how the performance increased as features were added; the model was then reduced by a backward elimination process to select the most compact model with the highest classification accuracy and the lowest number of features.

Subsequently, multivariate SVM classification models were created with a linear kernel, using the features obtained from the feature selection process by the GA, and refined by forward selection and backward elimination (see Table 4). The final SVM models have only two features each. To evaluate their performance and choose the most optimal model, they were subjected to cross-validation and blind tests.

The models in Table 4 were subjected to a five-fold cross-validation. Eighty percent of the FDS data were used for this process, which was separated to train and test for each of the models. The results obtained from training and testing the five-fold cross-validation of the ADvsMCI/CN-m1 model are shown in Table 5. This table reports the mean of the classification metrics for the five folds and the error that refers to the standard deviation of the obtained results.

For measuring the performance of the model in a new environment, the model was trained using the whole training dataset (80%) and subsequently validated by its performance on the blind test dataset (20%). The results of this blind test validation are presented in Table 6.

**Figure 6:**
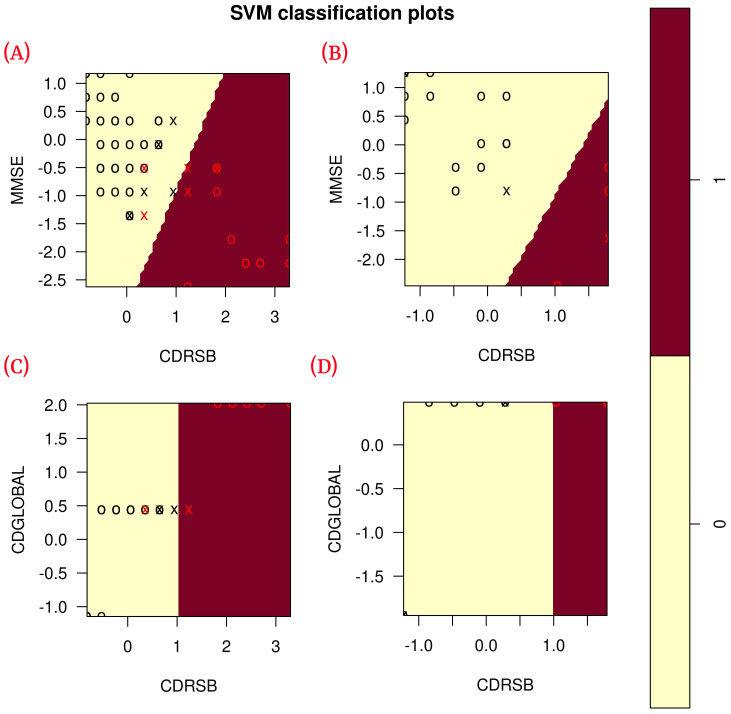
Correlation of the features of each model during training (80% training data) and blind test (20% blind subset). (**A**) Shows the correlation of ADvsMCI/CN-m1 model features in training; (**B**) shows the correlation of ADvsMCI/CN-m1 model features in the blind test; (**C**) shows the correlation of ADvsMCI/CN-m2 model features in training; (**D**) shows the correlation of ADvsMCI/CN-m2 model features in the blind test. In these plots, the model support vectors are represented with “X”; the points represented with “O” are the remaining data. The red color classifies the data where Alzheimer’s disease is present, while the black color classifies MCI/CN.

According to the results obtained in the blind test (Table 6), each of the classification metrics used to measure the performance of the models had a value of 1. To validate these results, the data were plotted using only the two features of each model to observe the correlation of the data (see Figure 6). The plots show that the data are linearly separable. This suggests that the use of a linear kernel in the SVM models for this study is appropriate.

## 5. Discussion

The proposed methodology demonstrates the effectiveness of using genetic algorithms and support vector machines systems for the classification of AD vs MCI/CN using multi-source information.

The methodology combined data (gene indexes and clinical and neuropsychological assessments) from the ADNI1 study in its baseline stage of 103 patients.

Subsequently, in the normalization stage, the features were scaled (z-score transformation) for use in patient classification. Using the features, the genetic algorithms generated 200 generations for 300 solutions in order to find the best performing multivariate model. As the models evolved, the average accuracy was plotted as depicted in Figure 4, the models reached their best performance within the first fifteen generations. Hence, 200 generations were defined as an optimal parameter, since no more generations were needed. The final model was refined using forward selection and backward elimination. The SVM models were constructed with the features obtained in Table 4.

The performance of the final model was evaluated using a cross-validation and a blind test to simulate a real-world scenario. The cross-validation model was trained and tested using 80% of the FDS dataset (see Table 4), while for the blind test, the model was trained using 80% of the FDS dataset and validated using the remaining 20% of the unseen data. The final SVM model to classify AD vs. MCI / CN was ADvsMCI/CN-m1, which obtained a sensitivity of 100% and a specificity of 100% in the blind test. The value of both validation metrics suggests that the model is robust. From over 900 features, the ADvsMCI/CN-m1 model included the following two features: MMSE and CDRSB.

The features included in the final model have been previously used as individual diagnostic features, such as the MMSE proposed by [18] and the CDRSB proposed by [24]. Our model combines individual prediction performance into a multivariate model capable of improving early diagnosis of AD. It was also observed that the CDRSB feature, which represents one of the scores of the CDR assessment, appears in both models, proving to be an important assessment in the classification of patients with some degree of dementia and healthy patients.

The model proposed by Zhang et al. [6] has a good performance for the classification of AD vs. CN patients. Nevertheless, in this model, most of the used features are extracted from medical images of patients (MRI and PET). The proposed model obtained in the present work avoids the use of features from medical images and obtains a performance as good as the one proposed by Zhang et al.; the latter empowers the proposed model, in this paper, with the advantage to be available in places where the access to medical images studies is limited.

Additionally, the proposed models in this study avoid the use of features obtained from laboratory tests to diagnose/classify patients between CN and MCI (or AD). This leads to a natural reduction of the required features. Our models show similar performance to the models proposed by Hassan et al. [26] and Stamate et al. [28], where the number of features is higher than fifteen. Using fewer features could be advantageous for patients who are vulnerable to laboratory tests or biopsies. It is hoped that the models proposed in this study are a viable alternative for this type of patient.

## 6. Conclusions

The proposed methodology in this study selects the most relevant features of AD data (gene indexes and clinical and neuropsychological assessment) through the use of genetic algorithms. These features were used to generate supervised classification algorithms with an SVM architecture. The efficiency of the generated models was evaluated by a cross-validation and a blind test, selecting the model with the highest sensitivity, specificity, and whose features exhibited a good performance during the blind test, for early detection of AD, between subjects with AD and MCI or CN subjects.

The novelty of this study is that it uses only non-imaging biomarkers, and yet a similar performance to those derived from medical images is reached. The obtained models integrated features that were previously individually validated by the research community. Therefore, the proposed multivariate study combines individual predictions into a more robust biomarker to detect early Alzheimer’s disease.

## 7. Future Work

For future work we, propose to combine features extracted directly from MRI and use them with the biomarkers obtained in this study to predict the likelihood of a CN patient evolving into an AD patient. We will also investigate the possibility of replacing those features that come from CSF analyses and blood-based metabolomics tests (since these analyses are considered invasive techniques) with features obtained from MRI and develop more robust ML models for the classification of patients with AD.

## Figures and Tables

**Table 1:** Examples of clinical and neuropsychological assessments and laboratory analysis for the diagnosis of AD considered in this work.

Assessments	Score Range	Score	Stages of Cognitive Function
MMSE	0–30	24–30	Normal cognitive
		19–23	Mild dementia
		10–18	Moderate dementia
		<9	Severe dementia
ADAS-Cog 11	0–70		Higher scores suggest greater severity of the cognitive symptoms of dementia
ADAS-Cog 13	0–85		Higher scores suggest greater severity of the cognitive symptoms of dementia
GDS	0–15	0–4	Normal
		5–8	Mild depression
		9–11	Moderate depression
		12–15	Severe depression
Global Deterioration Scale *	1–7	1	Normal cognitive
		2	Age associated memory impairment
		3	MCI
		4	Mild dementia
		5	Moderate dementia
		6	Moderately severe dementia
		7	Severe dementia
CDGLOBAL	0–3	0	No dementia
		0.5	Questionable dementia
		1	MCI
		2	Moderate cognitive impairment
		3	Severe cognitive impairment

* The Global Deterioration Scale assessment is not used by ADNI.

**Table 2:** Inclusion criteria.

Inclusion Criteria
Patients should have visit codes of baseline (bl), 12 months (m12), 24 months (m24) or 36 months (m36). Verify and check the participant roster ID (RID) to ensure that the measurements were from the same patient in the different datasets. In case of examinations and evaluation scales, only the final score was taken, avoiding redundant information. The age of the patients should be between 53 and 95 years at the enrolment date. No distinction of gender, education, ethnicity, race, marital status was performed. Patients should have biological, clinical and neuropsychological assessments. Patients with duplicated records were merged using the 1st non-empty record.

**Table 4:** Most important features for classification of patients with Alzheimer’s obtained through the GA.

Dataset Version	Model Name	Multivariate Model Type	Final Model Length	Features
D1	ADvsMCI/CN-m1	SVM	2	MMSE, CDRSB
D2	ADvsMCI/CN-m2	SVM	2	CDGLOBAL, CDRSB

**Table 5:** Performance metrics obtained by k-fold cross-validation of the ADvsMCI/CN-m1 model.

AD vs. MCI/CN			
Process	Metrics	Average	Error
Training	AUC	0.9079	0.0437
	Specificity	0.9882	0.0156
	Sensitivity	0.8276	0.0890
	Accuracy	0.9631	0.0185
Testing	AUC	0.8763	0.1024
	Specificity	0.9811	0.0307
	Sensitivity	0.7715	0.1957
	Accuracy	0.9433	0.0444

**Table 6:** Training metrics and blind test validation of SVM models.

Model Name				Model Name			
ADvsMCI/CN-m1				ADvsMCI/CN-m2			
Training (80%)		Blind Test (20%)		Training (80%)		Blind Test (20%)	
AUC	0.9231	AUC	1	AUC	0.9088	AUC	1
Specificity	1	Specificity	1	Specificity	0.9714	Specificity	1
Sensitivity	0.8461	Sensitivity	1	Sensitivity	0.8461	Sensitivity	1
Accuracy	0.9759	Accuracy	1	Accuracy	0.9518	Accuracy	1

Eighty percent of the dataset was used to train the model, and blind test validation was performed on the remaining 20%.

## Data Availability

The data presented in this study are available on request from corresponding author.

## Abbreviations

The following abbreviations are used in this manuscript:

AD	Alzheimer’s disease
ADAS	Alzheimer’s Disease Assessment Scale
ADAS-Cog 11	ADAS-Cognitive Subscale with 11 items
ADAS-Cog 13	ADAS-Cognitive Subscale with 13 items
ADNI	Alzheimer’s Disease Neuroimaging Initiative
AI	Artificial Intelligence
AUC	Area Under the Receiver Operating Characteristic Curve
bl	Baseline
CDGLOBAL	Clinical Dementia Rating Global Score
CDR	Clinical Dementia Rating
CDRSB	Clinical Dementia Rating sum-of-boxes score
CN	Cognitive Normal
CSF	Cerebrospinal Fluid
D1	Dataset 1
D2	Dataset 2
$F_{n}$	False Negative
$F_{p}$	False Positive
FAQ	Functional Activities Questionnaire
FDS	Filtered Dataset
GA	GALGO Genetic Algorithm
GDS	Geriatric Depression Scale
m12	12 Months
m24	24 Months
m36	36 Months
MCI	Mild Cognitive Impairment
ML	Machine Learning
MMSE	Mini-Mental State Examination
MRI	Magnetic Resonance Imaging
PET	Positron Emission Tomography
RID	Participant Roster ID
SVM	Support Vector Machine
$T_{n}$	True Negative
$T_{p}$	True Positive
